# Supplementary material for: A single mode of population covariation associates brain networks structure and behavior and predicts individual subjects’ age
Source: Commun Biol. 2021 Aug 5;4:943. doi: 10.1038/s42003-021-02451-0 (PMC8342440; doi:10.1038/s42003-021-02451-0)
Supplement: Supplementary file 3 — Description of Supplementary Files [file 42003_2021_2451_MOESM3_ESM.pdf]

Description of Additional Supplementary Files

File name: Supplementary Data 1
Description: Data reporting all values and error bars for Figures 1-5.
